# *Trans-*chalcone suppresses tumor growth mediated at least in part by the induction of heme oxygenase-1 in breast cancer

**DOI:** 10.1007/s43188-021-00089-y

**Published:** 2021-02-01

**Authors:** Tatiana Takahasi Komoto, Jaehak Lee, Pattawika Lertpatipanpong, Junsun Ryu, Mozart Marins, Ana Lúcia Fachin, Seung Joon Baek

**Affiliations:** 1 Laboratory of Signal Transduction, College of Veterinary Medicine and Research Institute for Veterinary Science, Seoul National University, 1 Gwanak-ro, Gwanak-gu, Seoul, 08826 Korea; 2 Biotechnology Unit, University of Ribeirão Preto, Av: Costábile Romano 2201, Ribeirão Preto, São Paulo 14096-900 Brazil; 3 Department of Otolaryngology-Head and Neck Surgery, Center for Thyroid Cancer, Research Institute and Hospital, National Cancer Center, Goyang, Korea; 4 Medicine School, University of Ribeirão Preto, Ribeirão Preto, São Paulo 14096-900 Brazil

**Keywords:** Heme oxygenase-1, *Trans*-chalcone, High content screening, Antibody array, Breast cancer, Xenograft

## Abstract

Despite intensive research efforts in recent decades, cancer remains a leading cause of death worldwide. The chalcone family is a promising group of phytochemicals for therapeutic use against cancer development. Naturally-occurring chalcones, as well as synthetic chalcone analogues, have shown many beneficial biological properties, including anti-inflammatory, antioxidant, and anti-cancer activities. In this report, *trans*-chalcone (TChal) was found to increase cell death in breast cancer cells, assessed using high content screening. Subsequently, using antibody array analysis, TChal was found to increase heme oxygenase-1 (HO-1) expression in TChal-treated breast cancer cells. Blocking of HO-1 by siRNA in breast cancer cells diminished the effect of TChal on cell growth inhibition. TChal-fed mice also showed less tumor growth compared to vehicle-fed mice. Overall, we found that TChal increases HO-1 expression in breast cancer cells, thereby enhancing anti-tumorigenesis. Our results suggest that HO-1 expression could be a potential new target of TChal for anti-tumorigenesis in breast cancer.

## Introduction

Breast cancer is classified in four different groups, according to its molecular characteristics: luminal A, luminal B HER-2 negative, luminal B HER-2 positive, and HER2 and basal-like tumors (basal-like or triple-negative) [1]. Luminal A and B are hormone-sensitive (estrogen receptor-positive or progesterone receptor-positive) with slightly invasive capacities and present a good clinical outcome. On the other hand, another subtype, HER-2 and basal-like breast cancer is insensitive to hormonal therapy, enhanced invasiveness, and aggressive metastatic capacity and presents a poor clinical outcome [2]. In addition, few systematic treatment options are available for the triple negative breast cancer (TNBC), and thus, intensive research is needed to identify specific targets and additional treatment options. The resistance to all classical therapeutic strategies led us to search for new drugs or alternatives that exhibit a better therapeutic response against TNBC. In this context, phytochemicals found in medicinal plants have been shown to inhibit tumorigenesis in experimental animals and/or exhibit potent anti-cancer properties [3]. Among those, chalcone has gained increasing attention due to its anti-cancer activity, including its diverse biological activities [4–6]. Chalcone may exist in two isomeric forms, *cis* and *trans*; the *trans*-chalcone (TChal) form is considered the more thermodynamically stable. TChal has been shown to exert cytotoxic activity against many cancer cells through multiple mechanisms, including cell cycle disruption, angiogenesis inhibition, tubulin polymerization inhibition, apoptosis induction, blockade of NF-κB signaling pathway, and the inhibition of cell cycle regulatory kinases [5–8]. In addition, TChal has been identified as an inhibitor of topoisomerase 2α (Top2A) [9], which is an important target of various anti-cancer drugs.

Of particular interest, TChal lacks genotoxic effects at the amino group of nucleic acid, which is found in most anti-cancer drugs. We have recently demonstrated that TChal exhibits anti-cancer activity mediated by an increase in tumor suppressor p53 [5, 6], and by binding to CRM1 protein, resulting in the accumulation of p53 in the nucleus [5]. Our group also demonstrated that TChal exhibits an anti-proliferative activity in in vitro assays against breast cancer cell lines (BT-20 and MCF-7), mediated by gene modulations of p53, AURKA, and Bcl2 [4, 8].

Heme oxygenase-1 (HO-1) is a phase II enzyme that responds to electrophilic stimuli, such as oxidative stress, cellular injury, and many diseases. HO-1 is elevated in various human malignancies, implicating its contribution to creating a tumor microenvironment which is appropriate for cancer cell growth, angiogenesis, and metastasis, as well as resistance to chemotherapy and radiation therapy [10]. However, augmented HO-1 expression in tumor cells can enhance cell death in various cancer cells [11–14]. Notably, HO-1 expression displays anti-cancer activities in breast cancer cells, as assessed by in vitro and in vivo assays, as well as human tumor samples [11]. It is also highly expressed by oxidative stimuli, such as UV irradiation, reactive oxygen species (ROS), growth factors, and inflammatory cytokines [10, 15]. Several studies have associated HO-1 expression with a poor prognosis [10], although others have pointed it out as an important protein responsible for decreasing the malignance of tumor progression and proliferation in several different types of cancers [14, 16–18]. Thus, HO-1 expression exhibits both anti- and pro-cancer activity, depending on the cell context and tumor type.

In the present study, we investigated the anti-cancer effects of HO-1 in human breast carcinoma cells. We demonstrated that the treatment of the BT-20 cell line (triple-negative) with TChal induces the expression of HO-1 protein. These cells become less sensitive to TChal when cells are treated with siRNA targeting HO-1 mRNA, suggesting that HO-1 expression plays a role in TChal-induced anti-tumorigenesis. Moreover, the anti-cancer activity of TChal was examined using BT-20 xenograft animal models and a reduction in tumor size and weight were observed, compared to the vehicle treated group. Overall, TChal treatment showed anti-cancer activity in vitro and in vivo, mediated by expression of HO-1 in breast cancer.

## Material and methods

### Cell lines and reagent

**Fig. 1:**
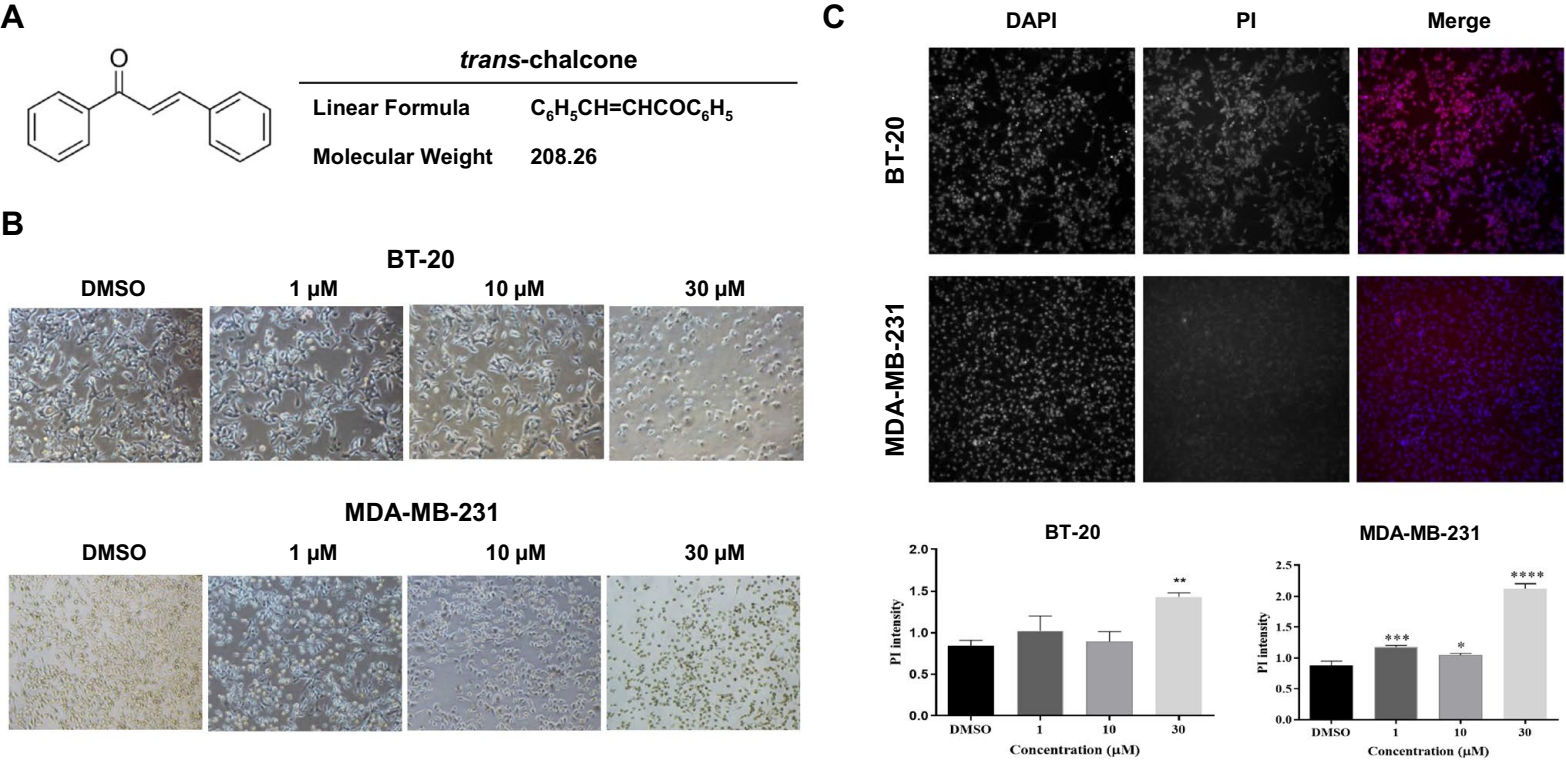
TChal increased cell death in breast cancer cells. **a** Tchal structure. **b** BT-20 and MDA-MB-231 cell lines were treated with TChal at the indicated concentrations for 24 h. Cell morphology was observed by microscope. **c** Cytotoxicity assay was performed on CX7 LZR using DAPI and PI staining. The top panel represents DMSO treated samples from BT-20 and MDA-MB-231 cells and the bottom graph represents quantification of PI staining after TChal treatment in the cells (*p < 0.05, **p < 0.01, ***p < 0.001)

Human triple-negative breast cancer cell lines BT-20 and MDA-MB-231 were purchased from Korea Cell Line Bank (Seoul, Korea). TPC-1 (thyroid cancer cell line) cells were generously provided by Dr. Gary Clayman (MD Anderson, TX, USA). BT-20 cells were cultured in RPMI medium, whereas MDA-MB-231 and TPC-1 cells were cultured in DMEM. These culture media were supplemented with 10% fetal bovine serum (FBS; Thermo Fisher Scientific, Waltham, MA, USA), 100 U/mL penicillin, and 100 mg/mL streptomycin (Gibco Life Technologies, Carlsbad, CA, USA). The cells were maintained at 37 °C under a humidified atmosphere containing 5% CO_2_. *TChal* (Fig. 1A) was purchased from Sigma-Aldrich (97% purity, Cat# 136123, St. Louis, MO, USA). Epoxomicin (Cat# E3652) was purchased from Sigma Aldrich. All culture media and trypsin were purchased from Gibco (Carlsbad, CA, USA). Tween-80 was purchased from MBcell (Seoul, Korea). The following antibodies were purchased from Santa Cruz Biotechnology (Dallas, TX, USA): anti-heme oxygenase 1 (sc-136961) and anti-β-actin (sc-47778).

### Cell toxicity assay by high content screening

The toxicity assay was performed using the CellInsight CX7 LZR High Content Screening (HCS) platform (Thermo Fisher Scientific, Waltham, MA, USA). Briefly, BT-20 and MDA-MB-231 cell lines were harvested and split at a concentration of 10,000 cells/well in 96-well culture plates in triplicate. After 24 h of incubation, TChal was added at 1, 10, and 30 µM for 24 h, with DMSO (0.1%) as a vehicle. The media were removed and the cells were washed twice with 1 × PBS, followed by the addition of 200 µL of propidium iodide working solution (333 ng/ml; Invitrogen, Carlsbad, CA, USA) into each well and an incubation period of 5 min at room temperature. The cells were washed with 1 × PBS twice and treated with 100% methanol for 15 min to permeabilize the cells, before washing again with PBS. Finally, 4′,6-diamidino-2-phenylindole (DAPI; Sigma-Aldrich, St. Louis, MO, USA) solution at 1 µg/mL was added into each well for 10 min and the cells washed with 1 × PBS for 3 times. The samples were analyzed using the CX7 LZR High Content Screening (HCS) platform with the cell toxicity assay method.

### Antibody array

Proteome Profiler Human XL Oncology (R&D Systems, Minneapolis, MN, USA) can identify 84 different human cancer-related proteins on the membrane. Experiments were performed according to the manufacturer’s protocol. Briefly, protein extraction was performed using RIPA buffer following the manufacturer’s instructions. Then, two membranes (one membrane for control lysates and another for TChal treated lysates) were blocked with array buffer for 1 h on a rocking shaker, and each cell lysate (200 μg) was added and incubated with membrane overnight at 4 °C. Then, both membranes were washed with 1 × wash buffer at least 3 times for 10 min, followed by the addition of the detection antibody cocktail solution for 1 h, before washing 3 times for 10 min. Streptavidin-HRP solution was then incubated for 30 min at room temperature and on a rocking platform shaker, followed by washing steps. The membranes were visualized by chemiluminescence detection using ECL Western Blot Detection Reagent (Amersham Biosciences, Piscataway, NJ) in Alliance Q9 mini (UVITEC, Cambridge, England, UK).

### Western blotting

Cells were washed with 1 × PBS and collected in RIPA buffer supplemented with proteinase inhibitors. The cells were kept on ice for 30 min followed by vortex for 5 min to promote the cell lysate. The cell lysate was then centrifuged at 13,000 g for 20 min at 4 °C. The supernatant was collected, and the total protein concentration was measured using a Pierce BCA Protein Assay Kit (Thermo Scientific, Rockford, IL, USA). Then, 30 μg of each protein was used in electrophoresis with 12% SDS-PAGE gel before being transferred to a nitrocellulose membrane. The membranes were blocked with TBST buffer (25 mM Tris, 3 mM KCI, 0.14 M NaCl, 0.05% Tween-20) containing 5% skim milk at room temperature for 1 h. Subsequently, the membranes were incubated with primary antibodies diluted in TBST-5% non-fat milk (1:1000) overnight at 4 °C. Afterwards, the membrane was washed with TBST and incubated with the secondary antibody for 2 h at room temperature, then washed with TBST. The proteins were visualized by chemiluminescence detection using ECL Western Blot Detection Reagent (Amersham Biosciences, Piscataway, NJ, USA) in Alliance Q9 mini (UVITEC).

### Quantitative real-time polymerase chain reaction

Total RNAs were extracted using TRIzol™ Reagent (Ambion®, Carlsbad, CA, USA). One μg of RNA was used as template to reversely transcribe into first-strand cDNA by Verso cDNA Synthesis Kit (AB1453B, Thermo Fisher Scientific, Waltham, MA, USA) using MiniAmp Plus Thermal Cycler (A37835, Applied Biosystems, Waltham, MA, USA). The primer pair for amplification of HO-1 was as followed: forward 5′-CCAGGCAGAGAATGCTGAGTTC-3′ and reverse 5′-AAGACTGGGCTCTCCTTGTTGC-3′. For normalization, expression of GAPDH was examined with the primer pair of: forward 5′-GAAGGTGAAGGTCGGAGTCA -3′ and reverse 5′-GACAAGCTTCCCGTTCTCAG-3΄. The relative level of each RNA was measured by real-time PCR using SYBR Green reagents (PowerUp™ SYBR™ Green Master Mix, A25741, Applied Biosystems, Thermo Scientific, USA) by QuantStudio™ 1 Real-Time PCR System (Applied Biosystems). The relative gene expression of HO-1 gene was analyzed by the comparative Ct (2^−ΔΔCt^) method.

### siRNA experiments

HO-1 siRNA was purchased from Santa Cruz Biotechnology (Dallas, TX, USA). Briefly, 3 × 10^5^ cells were harvested in a 6 well-plate and transfected with 30 nM of specific siRNA, or the transfection reagent as a control. The MISSION^®^ siRNA Transfection Reagent was used according to manufacturer’s instructions (Sigma-Aldrich). After transfection, the plate was incubated at 37 °C for 24 h. The transfected cells were then transferred to a 25-cm^2^ flask and maintained for 72 h to allow for cell recovery.

### Reverse transcription-polymerase chain reaction (RT-PCR)

The RNA was reverse transcribed using Verso cDNA Synthesis Kit (Thermo Fisher Scientific, Waltham, MA, USA), followed by PCR using GoTaq Green PCR Master Mix (Promega, Maidson, WI, USA) with heme oxygenase-1 (F: 5′-CTTCTTCACCTTCCCCAACA-3′ and R: 5′-GCTCTGGTCCTTGGTGTCAT-3′) and GAPDH (F: 5′-AATCCCATCACCATCTTCCAG-3′ and R: 5′-GAGCCCCAGCCTTCTCCAT-3′) primers. The thermal cycling conditions were as follows: initial denaturation at 94 °C for 2 min, followed by 40 cycles of 94 °C for 30 s, 59 °C for 30 s, and 72 °C for 45 s, before a final extension at 72 °C for 5 min. The products were electrophoresed on 1.5% agarose gel and photographed under UV light.

### Immunohistochemistry

Tissue blocks were fixed in 10% formalin and embedded in paraffin. Five-μm-thick sections were prepared for immunohistochemical analysis. Antigen retrieval was performed using a heat-induced epitope retrieval method with pressure cooker treatment in citrate buffer (Immunobioscience, Mukilteo, WA, USA) for 1 min. Endogenous peroxidase activity was blocked by treatment with 0.3% hydrogen peroxide (Sigma-Aldrich, St. Louis, MO, USA) in PBS for 1 h at room temperature. The bound antibody was performed using an Ultra-sensitive ABC Staining Kit (Thermo Fisher Scientific, Rockford, IL, USA), according to manufacturer’s recommendations. Mouse monoclonal antibody against HO-1 (Santa Cruz Biotechnology, Dallas, TX, USA) was used for treating the sections at 4 °C overnight. As a negative control, no primary antibody was used. For the detection of peroxidase (HRP) enzyme, the sections were developed using a 3,3′-diaminobenzidine tetrahydrochloride (DAB) substrate (ImmPACT^®^ DAB kit; VECTOR Laboratory, Burlingame, CA, USA) at room temperature for 45 s, before counterstaining with hematoxylin (VECTOR Laboratory, Burlingame, CA, USA) for 30 s. Coverslip mounted sections were observed using Pannoramic SCAN (3DHISTECH, Budapest, Hungary), a slide scanner. The optical density was measured using Image J 1.52a software, as previously described [19].

### Animal experiment

Animal care and procedures were approved by the Institutional Animal Care and Use Committees of Seoul National University (SNU-180119-2-6). Ten male nude mice of 6-week-old (25 g) were purchased from Japan SLC, Inc. (Hamamatsu, Japan) and used to evaluate the effect of TChal on tumor growth in vivo. The mice were housed in accordance with College of Veterinary Medicine policies from Seoul National University. The mice were inoculated with freshly harvested BT-20 cells at a concentration of 3 × 10^6^ cells/200 μl in 1 × PBS. Treatment with TChal was started after 2 weeks. The mice were randomly divided into three groups: control (3 mice), 5 mg/kg TChal (3 mice), and 50 mg/kg TChal (4 mice). TChal was diluted with 10% Tween-80 in saline and fed to the experimental mice 3 times a week (Monday, Wednesday and Friday) for 4 weeks by gavage, whereas the control mice were administrated using only the vehicle. After a 4-week treatment, the TChal administration was stopped because the tumor development was not detected at the higher concentration of TChal. Tumor measurements were performed with a caliper by measuring the largest diameter and its perpendicular length twice a week and the body weight was measured once a week. The tumor volume was calculated using the formula: V = length × width^2^. All mice were maintained on a standard chow diet and euthanized with a fill rate of 30% of the chamber volume per minute with 100% CO_2_ gas at the end of experiment.

### Statistical analysis

All experimental data were subject to analysis of variance (ANOVA), followed by the Bonferroni test. The level of statistical significance was set at *p* < 0.05.

## Results

### TChal enhances cell death in breast cancer cells

It has been reported that TChal induces cell death in several cancer cells [5, 6]. To investigate the effect of TChal on breast cancer cell growth, two different breast cancer cells, BT-20 and MDA-MB-231, were grown and treated with TChal (Fig. 1A). As shown in Fig. 1B, the cell morphology was significantly changed and the cell numbers were dramatically decreased in both types of cells after treatment with 30 µM of TChal for 24 h. Propidium iodide (PI) was administered to the cells and the PI intensity was measured using CellInsight CX7 LZR High Content Screening Platform (CX7 LZR). Consistent with Fig. 1B, TChal increased PI intensity in both cell lines, indicating that TChal induces cell death in two different types of breast cancer cells. These data are also supported by images captured by CX7 LZR (Fig. 1C).

### TChal increases HO-1 expression

**Fig. 2:**
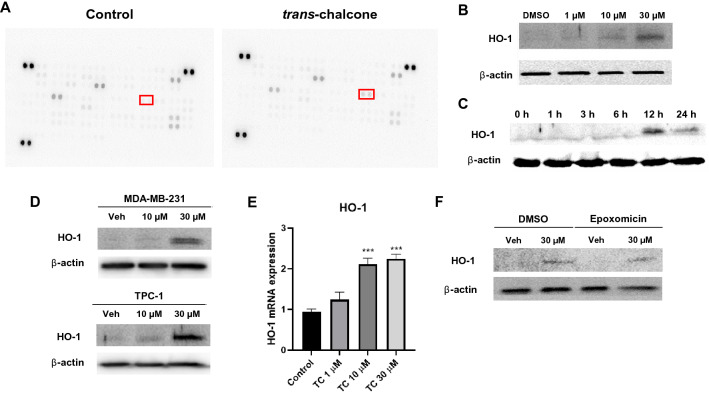
Increase of HO-1 expression by TChal. **a** Antibody array was performed to analyze the expression of 84 proteins on the membrane. Control or TChal-treated cell lysates from BT-20 cells were subjected to antibody array. The red box indicates HO-1, which was highly increased compared to the control. **b** Western blot analysis for the validation of the dose-dependent HO-1 expression in cancer cell lines treated with TChal at the indicated dose. β-actin was used as the internal control. **c** BT-20 cells were treated with 30 μM of TChal at different time points and cell lysates analyzed by western blotting. Equal loadings were measured by β-actin expression. **d** MDA-MB-231 and TPC-1 cells were treated with TChal and the cell lysates were subjected to western blotting. Equal loadings were measured by β-actin expression. **e** realtime PCR from BT-20 cells treated with different concentration of TChal for 24 h. ***p < 0.001. **f** Western blotting analysis for HO-1 in BT-20 cells treated with TChal for 1 h followed by the treatment of DMSO or epoxomicin for 24 h. Equal loadings were measured by β-actin expression

Antibody array was used to determine the relative expression levels of the selected human cancer-related proteins. To identify altered proteins affected by TChal, we performed an antibody array using DMSO- and 30 μM TChal-treated BT-20 cell lysates. The results from the antibody array showed one candidate protein, HO-1, out of a total of 84 cancer-related proteins (Fig. 2A). The expression of HO-1 on the antibody array membrane was estimated by Image J, and found to increase HO-1 expression by four-fold. To verify the antibody array data, western blot analysis was performed using TChal-treated cancer cells. As shown in Fig. 2B, C, TChal increased HO-1 expression in BT-20 breast cancer cells in a dose- and time-dependent manner. Interestingly, HO-1 seemed to increase to a maximum expression at 12 h, which then decreased after 24 h of treatment. Breast cancer MDA-MB-231 cells and human thyroid cancer TPC-1 cells were also examined to determine whether TChal increases HO-1 expression. As shown in Fig. 2D, TChal increased HO-1 expression in both MDA-MB-231 and TPC-1 cells in a dose-dependent manner. To determine whether TChal affects HO-1 RNA transcripts in BT-20 cells, total RNA was isolated and real-time PCR was performed with a specific primer derived from human HO-1 gene. As shown in Fig. 2E, the HO-1 transcript appeared to be increased in 10 and 30 μM TChal-treated samples, indicating that TChal may affect transcription level of HO-1 gene. Moreover, we examined whether TChal was able to prevent HO-1 degradation. We pre-treated BT-20 cells with DMSO or TChal for 12 h, followed by co-treatment with epoxomicin for 24 h. No significant HO-1 alteration was found (Fig. 2F). Finally, HO-1 protein was examined to determine whether it undergoes post-translational modifications, such as phosphorylation, which may alter its stability [20]. However, no phosphorylation by TChal was detected in HO-1 protein, as assessed by phosphor-tag SDS gel (data not shown).

### HO-1 blocking by siRNA results in decreased TChal effect on cell growth inhibition

**Fig. 3:**
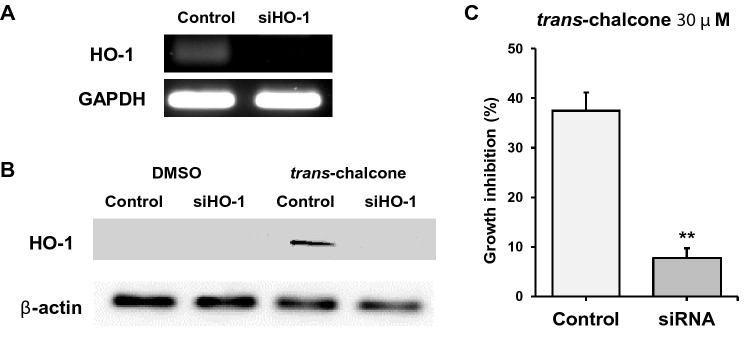
Analysis of HO-1 expression after siRNA transfection. **a** RT-PCR analysis using BT-20 cells transfected with HO-1 siRNA. The siRNA for HO-1 was transfected and the total RNA was isolated. RT-PCR for HO-1 was performed as described in the Methods section. GAPDH was used as the internal control. **b** Western analysis of HO-1 on BT-20 cells after HO-1 siRNA transfection. Equal loadings were measured by β-actin expression. **c** The toxicity assay on HO-1 siRNA-transfected BT-20 cells. After HO-1 siRNA transfection, the cells were tested for cell growth inhibition. Y-axis represents the percentage (%) of cell growth inhibition compared TChal to DMSO treated cells for 24 h. The data are representative of three independent experiments. **p < 0.01

To determine whether HO-1 plays a critical role in cell proliferation in the presence of TChal, siRNA for HO-1 was transfected and measured cell proliferation. The transfection of HO-1 siRNA blocked the expression of HO-1 transcript, as well as the protein expression (Fig. 3A, B). Based on the data, the transfected cells were used in a cell proliferation assay. As shown in Fig. 3C, the blocking of HO-1 in breast cancer cells exhibited less resistance to cell growth, compared to the control in the presence of TChal (Fig. 3C). These results suggest that the effect of TChal on breast cancer growth is mediated at least in part by HO-1.

### TChal suppressed tumor growth in nude mice

**Fig. 4:**
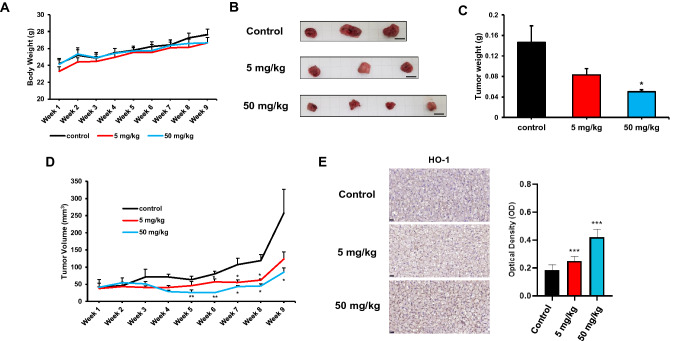
Analysis of effect of TChal in xenograft model. **a** The body weight of control and TChal-treated mice was measured for 9 weeks. **b** Tumors immediately after their removal. The scale bar located in the corner indicates 0.5 cm. **c** Tumor weight and **d** tumor volume was measured for 9 weeks. *p < 0.05, **p < 0.01, control vs TChal treatment. **e** The representative picture of immunohistochemistry. The bar in the left corner indicates 20 μm. The intensity was measured as described in method section and the graph was shown in the right

To determine whether TChal suppresses tumor growth in nude mice, actively growing BT-20 cells were subcutaneously injected into nude mice. When the tumor size reached around 30 mm^3^ (two weeks), mice were separated into three groups and TChal with 5 mg/kg and 50 mg/kg or vehicle was fed to mice by gavage. Nine weeks after the first treatment, the mice were sacrificed and the tumors were excised. As shown in Fig. 4A, the body weight was not changed between the groups. The weight of the tumors was measured, whereby TChal was found to reduce tumor weight in a dose-dependent manner (Fig. 4B, C). In addition, tumor volume was also significantly reduced in the presence of TChal, also in a dose-dependent manner (Fig. 4D). Finally, we measured HO-1 expression using tumor tissues, and found that HO-1 expression increased in TChal-treated mice compared to vehicle-treated mice (Fig. 4E). These data indicate that HO-1 expression plays a role in TChal-induced anti-tumorigenesis in breast cancer cells, assessed using a xenograft model.

## Discussion

Breast cancer is the most common cancer in women, accounting for 30% of all cases of cancer in women [21]. Breast cancer can be classified as either hormone-responsive (presence of ER, PR receptors, or both), HER-2 positive, or triple-negative. Among these different types, the triple negative type is the most difficult to treat and does not have any therapeutic target, unlike the other types [2]. Due to this poor prognosis, we decided to analyze the effect of TChal on BT-20 cells, a triple-negative cell line from the mammary gland.

Chalcones have been found to have many interesting biological properties, such as anti-tumor, anti-microbial, anti-inflammatory, and antioxidant activity. Specifically, TChal has been shown to exert potential anti-cancer activity by up-regulating the tumor suppressor protein p53 [5], and down-regulating the oncogenic protein AURKA [22]. A great advantage of TChal is that it does not present genotoxic effect [23].

The results obtained from the cell viability assay in this study is in accordance with those previously described by our group, in which TChal reduced the viability of BT-20 [4]. Similar results were found by other groups using breast cancer and osteosarcoma cells [6, 8]. In accordance with these findings, TChal has also been previously found to induce apoptosis, cell cycle arrest, and to inhibit migration, and invasion [5–8]. As the IC_50_ of TChal in BT-20 cells was identified [4]_,_ an antibody array assay was performed to identify the different modulated proteins between the treated and non-treated cell line as a result of treatment with TChal. Based on this assay, we found that HO-1 is highly expressed after treatment with TChal in a dose- and time-dependent manner in BT-20 cells, as well in the MDA-MB-231 and TPC-1 cell lines, indicating that this phenomenon happens in other cancer cells. A similar HO-1 induction was observed with another flavonoid, fisetin [24]. Thus, HO-1 may play a role in anti-cancer activity in response to various phytochemicals.

HO-1, also known as heat shock protein 32 (HSP32), is a stress-responsive protein and is considered an important adaptive survival response in stressed cells challenged with pro-inflammatory cytokines, oxidative stress, or UV irradiation [25]. In contrast, the role of HO-1 in tumorigenesis is controversial. Some reports suggest that HO-1 is up-regulated with malignancies, cancer progression, and therapy resistance in tumors, such as melanoma [26]. On the other hand, several studies have shown that HO-1 induces apoptosis and suppresses the proliferation and invasion of breast cancer cells and hepatocellular carcinomas [11–13]. In addition, sulforaphane, a chemopreventive agent, suppresses tumorigenesis by modulating HO-1 [27]. Furthermore, HO-1 is regulated by nuclear factor-erythroid-2-related factor 2 (NRF2), which seems to be a stress-responsive transcription factor, involved in encoding antioxidant enzymes and modulating inflammatory and immune responses, as well as protecting against neurodegenerative processes [28]. Phytochemicals with a α,β-unsaturated carbonyl moiety have been found to stimulate NRF2 signaling and consequently modulate cytoprotective enzymes, such as HO-1 [29]. Since TChal also displays a α, β-unsaturated carbonyl group, it is believed that TChal increases HO-1 expression, most likely by NRF2.

siRNA studies were performed to analyze the effect of TChal on HO-1 expression and revealed a reduced growth inhibition rate in silenced cells. Thus, our results suggest that TChal activity is at least in part mediated by HO-1 in BT-20 cells. Similar results were found for piperlongumine, an alkaloid whose activity has been associated with HO-1 expression [15]. After the suppression of HO-1, the ability of piperlongumine to induce apoptosis in MCF-7 cell line was inhibited [15]. Another group previously showed that HO-1 may be associated with the epithelial-mesenchymal transition (EMT), migration and proliferation inhibition of 4T1 cells (mouse mammary carcinoma cell line) [30].

In vivo assays were performed in order to validate the anti-tumor effect of TChal. Accordingly, our results indicate that TChal exerts anti-tumor activity against breast cancer. Treatment with 5 and 50 mg/kg TChal in mice was found to clearly inhibit the development of tumors, even at low TChal concentrations. These findings indicate that TChal could be a promising anti-cancer drug for treatment against breast cancer. To our knowledge, this is the first study to demonstrate the anti-tumor activity of TChal against breast cancer cells using an in vivo assay. TChal may modulate the expression of HO-1 at the protein level, thereby enhancing its anti-cancer activity.

In conclusion, the results presented demonstrate that HO-1 is an important factor regarding the effect of TChal against breast cancer development.

## References

[1] Huszno J, Kolosza Z (2019). Molecular characteristics of breast cancer according to clinicopathological factors. Mol Clin Oncol.

[2] Harbeck N, Gnant M (2017). Breast cancer. Lancet.

[3] Wenzel U, Kuntz S, Brendel MD, Daniel H (2000). Dietary flavone is a potent apoptosis inducer in human colon carcinoma cells. Cancer Res.

[4] Komoto TT, Bernardes TM, Mesquita TB, Bortolotto LFB, Silva G, Bitencourt TA, Baek SJ, Marins M, Fachin AL (2018). Chalcones repressed the AURKA and MDR proteins involved in metastasis and multiple drug resistance in breast cancer cell lines. Molecules.

[5] Silva G, Marins M, Chaichanasak N, Yoon Y, Fachin AL, Pinhanelli VC, Regasini LO, Dos Santos MB, Ayusso GM, Marques BC, Wu WW, Phue JN, Shen RF, Baek SJ (2018). Trans-chalcone increases p53 activity via DNAJB1/HSP40 induction and CRM1 inhibition. PLoS ONE.

[6] Silva G, Marins M, Fachin AL, Lee SH, Baek SJ (2016). Anti-cancer activity of trans-chalcone in osteosarcoma: involvement of Sp1 and p53. Mol Carcinog.

[7] Lamoke F, Labazi M, Montemari A, Parisi G, Varano M, Bartoli M (2011). Trans-chalcone prevents VEGF expression and retinal neovascularization in the ischemic retina. Exp Eye Res.

[8] Bortolotto LFB, Barbosa FR, Silva G, Bitencourt TA, Beleboni RO, Baek SJ, Marins M, Fachin AL (2017). Cytotoxicity of trans-chalcone and licochalcone A against breast cancer cells is due to apoptosis induction and cell cycle arrest. Biomed Pharmacother.

[9] Da Silva JG, Recio Despaigne AA, Louro SR, Bandeira CC, Souza-Fagundes EM, Beraldo H (2013). Cytotoxic activity, albumin and DNA binding of new copper(II) complexes with chalcone-derived thiosemicarbazones. Eur J Med Chem.

[10] Nitti M, Piras S, Marinari UM, Moretta L, Pronzato MA, Furfaro AL (2017). HO-1 induction in cancer progression: a matter of cell adaptation. Antioxidants (Basel).

[11] Gandini NA, Alonso EN, Fermento ME, Mascaro M, Abba MC, Colo GP, Arevalo J, Ferronato MJ, Guevara JA, Nunez M, Pichel P, Curino AC, Facchinetti MM (2019). Heme oxygenase-1 has an antitumor role in breast cancer. Antioxid Redox Signal.

[12] Zou C, Zou C, Cheng W, Li Q, Han Z, Wang X, Jin J, Zou J, Liu Z, Zhou Z, Zhao W, Du Z (2016). Heme oxygenase-1 retards hepatocellular carcinoma progression through the microRNA pathway. Oncol Rep.

[13] Lin CW, Shen SC, Hou WC, Yang LY, Chen YC (2008). Heme oxygenase-1 inhibits breast cancer invasion via suppressing the expression of matrix metalloproteinase-9. Mol Cancer Ther.

[14] Andrés NC, Fermento ME, Gandini NA, Romero AL, Ferro A, Donna LG, Curino AC, Facchinetti MM (2014). Heme oxygenase-1 has antitumoral effects in colorectal cancer: Involvement of p53. Exp Mol Pathol.

[15] Lee HN, Jin HO, Park JA, Kim JH, Kim JY, Kim B, Kim W, Hong SE, Lee YH, Chang YH, Hong SI, Hong YJ, Park IC, Surh YJ, Lee JK (2015). Heme oxygenase-1 determines the differential response of breast cancer and normal cells to piperlongumine. Mol Cells.

[16] Hill M, Pereira V, Chauveau C, Zagani R, Remy S, Tesson L, Mazal D, Ubillos L, Brion R, Asghar K, Mashreghi MF, Kotsch K, Moffett J, Doebis C, Seifert M, Boczkowski J, Osinaga E, Anegon I (2005). Heme oxygenase-1 inhibits rat and human breast cancer cell proliferation: mutual cross inhibition with indoleamine 2,3-dioxygenase. FASEB J.

[17] Caballero F, Meiss R, Gimenez A, Batlle A, Vazquez E (2004). Immunohistochemical analysis of heme oxygenase-1 in preneoplastic and neoplastic lesions during chemical hepatocarcinogenesis. Int J Exp Pathol.

[18] Paez AV, Pallavicini C, Schuster F, Valacco MP, Giudice J, Ortiz EG, Anselmino N, Labanca E, Binaghi M, Salierno M, Martí MA, Cotignola JH, Woloszynska-Read A, Bruno L, Levi V, Navone N, Vazquez ES, Gueron G (2016). Heme oxygenase-1 in the forefront of a multi-molecular network that governs cell–cell contacts and filopodia-induced zippering in prostate cancer. Cell Death Dis.

[19] Crowe AR, Yue W (2019). Semi-quantitative determination of protein expression using immunohistochemistry staining and analysis: an integrated protocol. Bio-protocol.

[20] Nishi H, Shaytan A, Panchenko AR (2014). Physicochemical mechanisms of protein regulation by phosphorylation. Front Genet.

[21] Siegel RL, Miller KD, Jemal A (2019). Cancer statistics. CA Cancer J Clin.

[22] Komoto TT, Bernardes TM, Mesquita TB, Bortolotto LFB, Silva G, Bitencourt TA, Baek SJ, Marins M, Fachin AL (2018). Chalcones repressed the AURKA and MDR proteins involved in metastasis and multiple drug resistance in breast cancer cell lines. Molecules.

[23] Lima DC, Vale CR, Veras JH, Bernardes A, Perez CN, Chen-Chen L (2017). Absence of genotoxic effects of the chalcone (E)-1-(2-hydroxyphenyl)-3-(4-methylphenyl)-prop-2-en-1-one) and its potential chemoprevention against DNA damage using in vitro and in vivo assays. PLoS ONE.

[24] Tsai CF, Chen JH, Chang CN, Lu DY, Chang PC, Wang SL, Yeh WL (2018). Fisetin inhibits cell migration via inducing HO-1 and reducing MMPs expression in breast cancer cell lines. Food Chem Toxicol.

[25] Chiang SK, Chen SE, Chang LC (2018). A dual role of heme oxygenase-1 in cancer cells. Int J Mol Sci.

[26] Was H, Cichon T, Smolarczyk R, Rudnicka D, Stopa M, Chevalier C, Leger JJ, Lackowska B, Grochot A, Bojkowska K, Ratajska A, Kieda C, Szala S, Dulak J, Jozkowicz A (2006). Overexpression of heme oxygenase-1 in murine melanoma: increased proliferation and viability of tumor cells, decreased survival of mice. Am J Pathol.

[27] Keum Y-S, Yu S, Chang PP-J, Yuan X, Kim J-H, Xu C, Han J, Agarwal A, Kong A-NT (2006). Mechanism of action of sulforaphane: inhibition of p38 mitogen-activated protein kinase isoforms contributing to the induction of antioxidant response element-mediated heme oxygenase-1 in human hepatoma HepG2 cells. Can Res.

[28] Loboda A, Damulewicz M, Pyza E, Jozkowicz A, Dulak J (2016). Role of Nrf2/HO-1 system in development, oxidative stress response and diseases: an evolutionarily conserved mechanism. Cell Mol Life Sci.

[29] Zhang DD, Lo SC, Cross JV, Templeton DJ, Hannink M (2004). Keap1 is a redox-regulated substrate adaptor protein for a Cul3-dependent ubiquitin ligase complex. Mol Cell Biol.

[30] Li Q, Liu Q, Cheng W, Wei H, Jiang Fang WE, Yu Y, Jin J, Zou C (2019). Heme oxygenase-1 inhibits tumor metastasis mediated by notch1 pathway in murine mammary carcinoma. Oncol Res.

